# Cannabis, Impaired Driving, and Road Safety: An Overview of Key Questions and Issues

**DOI:** 10.3389/fpsyt.2021.641549

**Published:** 2021-08-19

**Authors:** Bruna Brands, Patricia Di Ciano, Robert E. Mann

**Affiliations:** ^1^Health Canada, Ottawa, ON, Canada; ^2^Institute for Mental Health Policy Research, Centre for Addiction and Mental Health, Toronto, ON, Canada; ^3^Department of Pharmacology and Toxicology, University of Toronto, Toronto, ON, Canada; ^4^Campbell Family Mental Health Research Institute, Toronto, ON, Canada; ^5^Dalla Lana School of Public Health, Toronto, ON, Canada

**Keywords:** cannabis, driving, impaired-driving, medical, non-medical

## Abstract

The road safety impact of cannabis has been a topic of much discussion and debate over the years. These discussions have been revitalized in recent years by initiatives in several jurisdictions to legalize non-medical cannabis. Canada became the second country to legalize non-medical cannabis use in October, 2018, preceded by Uruguay in December 2013. Road safety concerns were key issues in the Canadian government's deliberations on the issue. In this paper, we identify several key questions related to the impact of cannabis on road safety, and provide a consideration of the relevant literature on these questions. These questions cover several perspectives. From an epidemiological perspective, perhaps the central question is whether cannabis use contributes to the chances of being involved in a collision. The answer to this question has evolved in recent years as the ability to conduct the relevant studies has evolved. A related question is the extent to which cannabis plays an important role in road safety, and recent research has made progress in estimating the collisions, injuries, and deaths that may be attributed to cannabis use. Several questions relate to the behavioral and pharmacological effects of cannabis. One central question is whether cannabis affects driving skills in ways that can increase the chances of being involved in a collision. Another important question is whether the effects of the drug on the driving behavior of medical users is similar to, or different from, the effects on non-medical users and whether there are sex differences in the pharmacological and behavioral effects of cannabis. Other important questions are the impact of tolerance to the effects of cannabis on road safety as well as different routes of administration (e.g., edibles, vaped). It remains unclear if there is a dose-response relationship of cannabis to changes in driving. These and other key questions and issues are identified and discussed in this paper.

## Introduction

Cannabis came under national and international regulation in the twentieth century [e.g., (1)], and for most of that century medical and non-medical use of the drug was illegal in most developed countries. However, in the latter half of the twentieth century, many began to question the wisdom of prohibiting cannabis use [e.g., (2)] at the same time as research began to appear suggesting that cannabis may have legitimate medical uses (3). As well, others examining the harms created by cannabis in comparison to other drugs observed that cannabis did not appear to be as harmful as the legal drugs tobacco and alcohol, further questioning the legitimacy of prohibition (4). Thus, toward the end of the twentieth century several jurisdictions had authorized cannabis use for medical purposes, and in the early years of the twenty-first century several jurisdictions have legalized cannabis for non-medical use (5). However, one area where evidence provides strongest support of an adverse impact of cannabis is its impact on traffic safety (6), and thus, the movement to legalization has brought increasing interest in the road safety impact of cannabis and how any negative effects might be addressed.

Understanding and addressing road safety problems resulting from cannabis is a complex challenge that requires insights from a variety of research fields, including pharmacology, epidemiology, and behavioral sciences, among others. While impaired driving is an international issue that affects every country (7), our understanding of driving under the influence of cannabis (DUIC) has largely been derived from studies conducted in higher income countries. However, important recent studies are confirming that DUIC is a road safety issue in lower income countries as well (8, 9). Studies have also focused on the impact of cannabis on drivers of automobiles. Nevertheless, it is clear that other road users, such as pedestrians and cyclists, may be under the influence of cannabis (9, 10), and the first study to assess the impact of cannabis on the injury risk of cyclists provided some evidence that cannabis may increase their risks of injuries resulting from crashes (11).

The increased attention to the road safety impact of cannabis in recent years has expanded our understanding of this topic, nevertheless many questions remain. In this paper we provide an overview of key questions related to cannabis and road safety, and point to current information on each topic as well as significant issues that remain to be addressed. We begin with a consideration of questions arising from epidemiological studies of DUIC. We then consider questions arising from the pharmacology of cannabis and its effects on behavior, including driving behavior.

### Does Cannabis Increase Collision Risk?

Perhaps the central question that must first be addressed is whether or not drivers who have recently used cannabis are at increased likelihood of being involved in a collision. Nevertheless, it is only relatively recently that a consensus on this question has emerged. Early evidence on this topic was not conclusive, and in a 1999 review Bates and Blakely concluded that, “There is no evidence that consumption of cannabis alone increases the risk of culpability for traffic crash fatalities or injuries for which hospitalization occurs, and may reduce those risks [(12), (p. 231)].” However, their conclusions were based on a relatively small number of studies addressing questions that were methodologically challenging. Since then, much more research on this topic has appeared, employing more rigorous methodologies, and current information suggests a different conclusion. Three meta-analyses have been published that conclude that the acute use of cannabis does increase collision risk (13–15), although the extent of the increase differed across studies. Even so, studies continue to appear suggesting that more needs to be understood in terms of how cannabis might increase collision risk (16). As well, research has yet to establish the characteristics of cannabis-involved collisions, or the impact of cannabis on injury severity resulting from collisions (17, 18).

### Who Drives Under the Influence of Cannabis?

If cannabis increases collision risk, it is important for prevention purposes to understand the people who report DUIC. Some factors have been identified that appear to predict an increased likelihood of DUIC. These include being an adolescent or young adult (19–21), experiencing cannabis-related problems (22), and possibly being a medical cannabis user [as opposed to a recreational cannabis user (23)]. As well, risk taking propensities, including reporting driving after drinking, also appear to be associated with increased likelihood of DUIC (21, 22). However, it should be noted that most research on the characteristics of people who report DUIC is derived from studies conducted where or when use was illegal, and thus it is possible that these characteristics may differ in legalized cannabis environments (24).

### Does Cannabis Play an Important Role in the Road Safety “Big Picture”?

For many years, the impact of cannabis on road safety received relatively little attention, in part because attention of researchers was focused on other topics such as alcohol and traffic safety. However, as noted the topic of cannabis and road safety has received much more attention, to the point where some initial estimations of the role of cannabis in the larger road safety picture could be estimated, as has been done with alcohol (25). Wettlaufer et al. (26) were able to estimate the total numbers of fatalities, injuries, and collisions, as well as the estimated social costs, that could be attributed to driving under the influence of cannabis in Canada in 2012, based on estimates of the impact of cannabis on the odds of collision involvement [e.g., (13)] and the prevalence of DUIC, among other things (26). They estimated that in Canada in 2012, cannabis attributable collisions accounted for 75 deaths and 4,407 injuries, with an additional 7,794 individuals involved in property-damage only collisions, which resulted in estimated costs of over $1 billion (CDN), using willingness to pay methods. The largest portion of casualties and costs resulted from collisions involving young drivers, since they are most likely to drive under the influence of cannabis.

### How Does Cannabis Affect Driving Behavior in Ways That Influence Collision Risk?

In order to fully understand the road safety impact of cannabis, and identify appropriate responses to that impact, it is important to understand how cannabis affects driving behavior. A small number of studies have employed observational studies of drivers given cannabis to consume (27), but these studies present important challenges to implement and carry out safely. One safe and objective means of studying the effects of psychoactive drugs on driving in the laboratory is with a driving simulator. Simulator research provides a controlled means of studying a variety of variables that cannot be easily controlled in the real world. For example, comparisons of driving in different situations (day vs. night, rural vs. urban) are challenging in on-the-road situations. Perhaps most important for the study of psychoactive drugs, the safety of the simulator cannot be understated, and studies of the effects of psychoactive substances with known impairing effects are likely best conducted in a safe, controlled, environment.

## Laboratory Studies

Laboratory studies of the effects of cannabis on driving date back many decades. The findings of these early studies need to be considered within the context of the changing landscape of non-medical cannabis use. Over time, it is known that the potency of recreational cannabis has steadily increased, and cannabis now has upwards of 10% THC, with some potency estimates as high as 19–22% (28–30). Thus, the utility of some of the early research using low potencies of THC (e.g., 1–3%) is limited in our modern world. For this reason, we will focus this review on studies published on or after the year 2000. Throughout, potencies of cannabis are provided for the cited studies.

The present overview of laboratory studies of the effects of cannabis on simulated driving will be structured into several themes. The first section will focus on findings using different routes of administration of cannabis. Next, claims of tolerance to the impairing effects of cannabis in frequent users will be evaluated. Differences in the effects of cannabis on driving in males and females will be explored, as will dose-response relationships of cannabis to driving and THC. Finally, the effects of medical cannabis on driving will be reviewed.

### Different Routes of Administration

#### Smoking

Smoking remains the most common route of administration (31), and thus, it has appropriately been the route of administration most frequently studied in simulator studies. It is estimated that about 84% of people who report past year use of cannabis have smoked their cannabis (31). Simulator studies have generally used between 1.77% THC and 6.7% THC in the studies, or 10–30 mg of THC in a cigarette. These represent low doses.

With respect to the use of cannabis, simulator studies have found important changes in driving after use of smoked cannabis. Consistent with epidemiological data (32, 33), one study found an increase in collisions after use of cannabis (34). Perhaps the most consistent finding with smoked cannabis is an increase in Standard Deviation of Lateral (SDLP), a measure of weaving (35–37). Despite increases in SDLP no effects on inappropriate lane crossings (37, 38) or lane position (34) were found [but see: (39)]. Similarly, steering angle was not affected (36), while steering control was decreased at a low dose in one study (39) but not another (34). Another finding that has been reported is changes in measures of speed. In general, cannabis decreases speed (36, 39–41) and speed variability (39). Measures of reaction time are also increased (36, 39). However, no effects on brake latency were found in other studies (42, 43). Increased headway (36) was also reported. Finally, one study reported an increase in “penalty points” after 900 micrograms/kg of THC in cigarettes. Penalty points were assigned for various driving infractions (44).

#### Vaporized

Vaping has recently received a great deal of media attention, due to concerns over the safety of this route of administration. In the real world, about 27% of cannabis users vape using a pen or e-cigarette, while 15% use a vaporizer (31). At the time of writing, only a few studies examined the effects of vaped THC on simulated driving. In one study, participants vaped 11% THC. Consistent with the smoked route, vaped THC increased SDLP (45), when required to follow and maintain a given distance to the car ahead (46). In this same task, no effect on headway was found. When instructed to follow GPS segments on highway and rural roads, no effects of THC were found. In another study, participants vaped 12.9% THC and the only effect observed was on crash risk at 1, 3, and 5 h post-cannabis (47). No effects were seen on braking reaction times, steering reaction time, lane-keeping speed control, intersection crossing, vigilance, obstacle avoidance accuracy, and obstacle avoidance crash risk. Thus, the effects of vaped THC seem to vary depending on the task parameters, but clearly more research is required.

#### Oral

Approximately 46% of people who use cannabis consume their cannabis in food (31). Thus, it is of interest to determine the effects of cannabis edibles on driving. At present, we are not aware of any published studies of the effects of cannabis edibles on simulated driving. However, clues as to the impact of the oral route of administration on driving can be obtained from the effects of synthetic THC, in the form of dronabinol. In one study with a crossover design with two doses of oral dronabinol (10 and 20 mg) or placebo, participants drove at a constant speed on a rural road or behind a lead car in a car-following task. Dronabinol increased SDLP, but did not affect speed measures (48). In another study with oral dronabinol (0, 10, and 20 mg), SDLP was also increased, as was reaction time (49). There were no effects on gain or coherence. In this study, gain was defined as the degree of reaction to speed changes to the car in front, while coherence was the degree to which the patterns of speed changes for lead and following car corresponded.

### Frequent vs. Occasional Users

The repeated use of cannabis can, under certain conditions, lead to tolerance to some of its effects (50, 51). However, recent meta-analyses of the effects of repeated cannabis in humans have yielded equivocal conclusions. For example, one meta-analysis concluded that there is tolerance to some cognitive measures, such as changes in EEG and to the intoxicating effects of THC (50). Findings from studies of psychomotor function, attention and memory were mixed (51).

Findings of tolerance to the effects of cannabis on driving are also mixed. At present, there have been very few studies of the effects of frequent cannabis use on simulated driving. In one study, driving impairments following a low dose (1.8–3% THC) of smoked cannabis were worse in regular cannabis users compared to non-regular users (52), suggesting that there was no tolerance of the effects of cannabis on driving. In another study, weaving was more evident in occasional users, as compared to regular users after oral synthetic THC (dronabinol) (48), suggesting that regular users may become tolerant to the effects of cannabis. SDLP was also greater in occasional users in another study that administered 10 or 30 mg of THC in a smoked cigarette (35). Further studies are warranted to investigate the effects of repeated cannabis use on driving following smoked recreational doses of cannabis.

It is of note that some recent studies found that heavy, chronic users of cannabis were impaired on the driving simulator, even in the absence of acute intoxication. In one study (53), compared to healthy controls, chronic cannabis users hit more pedestrians, missed more stop signs, made fewer stops at red lights, drove faster, and made more centerline crossings. In another study, chronic cannabis users had slower reaction times, deviated less in their speed and drove slower than the car ahead (54). They did not differ in lane position, speed, car following, off-road accidents, collisions and pedestrians hit. Clearly, tolerance to the effects of cannabis on driving has road safety implications and more research is needed.

### Effects of Sex

Sex appears to have important relationships with cannabis dependence. For example, more men than women use cannabis (55), although this gap is narrowing. Despite this, women show a greater progression to dependence than men (56), which may be related to the more severe withdrawal from cannabis that is reported by women (57). In our preliminary study with recreational users, we investigated sex differences in physiological and subjective effects (58). We found that females and males reported similar subjective and cognitive effects of cannabis, despite the observation that males had almost twice the blood level of THC as compared to females, and that males smoked more of the cannabis cigarette than females.

In one study which directly compared the effects of cannabis on driving in males to females (40), it was found, consistent with our findings, that more men than women finished the entire cigarette with 2.9% THC. Despite this, women rated themselves as feeling higher than males, and males were less sleepy than females. Thus, it appears that females were more sensitive to the subjective effects of cannabis than males. Despite this, there were no differences in measures of driving. A related paper by the same group also found no sex differences in cognitive effects of cannabis, despite the fact that males smoked more than females (59).

## Thc And Driving

As discussed above, there is good consensus that cannabis increases the risk of collision and alters SDLP and sometimes speed in simulator studies. Given the risks of using cannabis prior to driving, some jurisdictions have adopted limits on blood levels of delta-9-tetrahydrocannabinol (THC) while driving. These limits are generally in the range of 2–5 ng/ml. Given the success of limits on breath alcohol levels, it would be of interest to determine cut-off levels of THC for driving. A recently published paper by Arkell et al. (60) concluded that there is a poor and inconsistent relationship between levels of THC in biological fluids and degree of impairment. This led them to conclude that *per se* limits cannot discriminate between impaired and unimpaired drivers. They also concluded that more research is needed. It should be noted that he study that they used to test their hypothesis involved occasional users.

From the studies published thus far, it appears that there is a dose-response relationship of THC to changes in driving. For example, in one study, it has been reported that SDLP was increased by THC (46). Another study found that increasing blood THC was associated with decreased mean speed and increased following distance, with effects being observed at THC levels as low as 2 ng/ml (61). Changes in speed and reaction time were also dose-dependent (36, 39, 52), but THC was not measured in these studies. In another study, impairments, measured as “penalty points” were seen at 15 ng/ml of THC in blood (44). Nevertheless, more research is needed to gain a better understanding of the relationship between blood levels of THC and alterations in driving behavior (60).

We have recently published a paper that explored the relationship of THC to driving (41). In this study, participants drove a simulator at 30 min, 24 and 48 h after smoking a cannabis cigarette with 12.5% THC or placebo, in a parallel groups design. THC in blood was analyzed throughout the session, and participants were divided into high THC groups and low THC groups based on a median split of THC in the blood. We found that the high THC group drove significantly slower 30 min after smoking, as compared to placebo. Under dual task conditions, both the low THC and high THC groups drove slower. With respect to SDLP, the high THC group was different from placebo at 30 min and 48 h after smoking cannabis under single task conditions. There was no effect of cannabis on SDLP under dual task conditions. Thus, important evidence for a dose-response relationship was found, indicating that THC levels in blood may be related to changes in driving.

### THC and Driving After the Medical Use of Cannabis

Many jurisdictions have adopted *per se* laws in an attempt to curb DUIC, regardless of whether cannabis is used recreationally or for medical reasons. Despite these types of laws, courts in some jurisdictions of the world have seen challenges, in which medical users assert that their driving is unaffected by cannabis use due to the development of a drug tolerance associated with more frequent use. However, to date, there has been little research attention specifically on the effects of medical cannabis use on driving. This gap in the literature is dangerous given the prevalence of medical use of cannabis: 13% of cannabis users in a recent Canadian survey indicated that they used cannabis for medical purposes (31), and only half of these had a medical authorization (62). It is estimated that over half of those who use cannabis for medical purposes have driven within 2 h of using cannabis (63, 64) and most of these users indicated that there was “no risk” or “slight risk” of driving after the medical use of cannabis (64). Indeed, *many believe that therapeutic cannabis users are able to drive safely after using the drug* (65).

In another of our studies, we investigated the effects of cannabis on simulated driving in participants who use cannabis for medical reasons (64). In this study, we found that, consistent with our findings from recreational users, mean speed was decreased. Decreases in speed were similar to those observed in the recreational users, suggesting that medical users do not become tolerant to the effects of cannabis on driving. Thus, our findings suggest that “cannabis is cannabis” and it produces impairments in driving, regardless of whether it used for recreational or medical purposes. In our published study, we also observed that blood levels of THC were increased after smoking cannabis for medical reasons. The levels of THC in the blood did not show any evidence of tolerance when compared to THC levels after smoking cannabis for recreational purposes. Thus, medical users of cannabis should exercise caution in driving after the use of cannabis for medical purposes.

### What Levels of Cannabis in the Body Are Consistently Associated With Impairment?

There are a number of sources of information relevant to the question of what levels of cannabis in the body are associated with impairment. As well the answer to this question is important for considerations of whether or not to introduce a *per se* level, or legal limit, for cannabis and at what level. Laboratory studies of the effects of cannabis on basic behavioral and cognitive tasks, such as simple and choice reaction time, tracking ability, and memory, show that performance on a variety of measures is affected by smoking cannabis or consuming it in other ways (66–68). Nevertheless, the level at which impairment can be reliably seen has been a more controversial topic. Some have suggested that the effects of cannabis are not sufficiently dose-related to permit the identification of these levels. While the number of studies showing impairing effects of cannabis on driving-related skills is increasing, it is true that far fewer studies address the issue of whether that impairment is related to dose or level of THC in the body. Nevertheless, in laboratory studies of cognitive and behavioral effects there is evidence that the effects of cannabis increase as the dose consumed or level of THC in blood increases (69, 70). Evidence that effects of cannabis on driving simulator performance and collision risk increase as dose consumed and levels in the body increase has also been reported (71).

Based on these observations, *per se* levels for cannabis supported by evidence have been proposed. These levels have been based on literature reviews or meta-analyses of efforts to identify comparable levels of impairment caused by THC and specific levels of alcohol in the body. Grotenhermen et al. (72) proposed that serum levels of cannabis between 7 and 10 ng/ml caused levels of impairment that were comparable to Blood Alcohol Levels (BALs) of 0.05%. Vindenes et al. (73) suggested that 3 ng/ml of THC in whole blood was comparable to a BAL of 0.05%, and that 9 ng/ml of THC in whole blood was comparable to a BAL of 0.12% BAC (73). In the comprehensive European DRUID project, investigators concluded that 3.8 ng/ml of THC in serum was equivalent to a BAL of 0.05% (74).

## Cannabidiol and Driving

Cannabis contains a number of ingredients, and apart from THC, cannabidiol (CBD) has received the most widespread research interest. The proportion of THC to CBD may vary in cannabis, and thus it may be possible to develop strains of cannabis that reduce the risk of THC to driving. Within this context, a recent review of the effects of THC:CBD oromucosal sprays on driving did not find an impairment in driving in patients with multiple sclerosis who were using CBD:THC oromucosal sprays to treat their symptoms (75). Specifically, 80–90% of respondents reported no change in driving ability as a result of use of the THC:CBD oromucosal spray. This suggests that CBD may not impact driving ability. Although compelling, the studies here were observational in nature and focused on THC:CBD combinations; no experimental studies of the effects of CBD alone on driving have been conducted.

A recent study examined the possibility that CBD may impact the effects of THC on driving. In this study with a crossover design (45), participants vaped 11% THC, THC/CBD (11% THC, 11% CBD) or placebo. The THC/CBD condition increased SDLP to the same extent as THC alone, suggesting that CBD does not affect THC-induced changes in driving. Further, there were no effects of any of the conditions on headway, but the CBD/THC condition had greater standard deviation of headway than placebo. Thus, there is some evidence that combinations of THC and CBD may negatively impact driving. It should be noted that this study used the vaped route, and CBD is often taken orally, thus more studies are warranted, especially given the findings from observational studies and investigation of CBD and THC combinations on cognition. A subsequent study investigate the effects of different strains of cannabis with different proportions of THC and CBD on driving (76). They found that SDLP was increased after THC-dominant strains of cannabis, or after cannabis with equal amounts of THC and CBD, but not after CBD-dominant strains. This suggests that CBD does not impact driving and also does not reverse the deficit in driving observed after cannabis.

## How Long Do the Effects of Cannabis on Driving Behavior Last?

One concern about the effects of cannabis on driving behavior has been the length of time that impairment might last. This issue has often been confused with the length of time that THC or its metabolites can be measured in blood or other bodily fluids. Trace amounts of THC or its metabolites may be detected for days or even weeks following cessation of use (77). However, simple presence of THC or its metabolites does not mean that driving performance measures are impaired. Instead, similar to the above discussion of the levels of THC at which impairment is observed, the practical question is how long does impairment last following consumption of cannabis.

Some early studies suggested that residual effects of a dose of cannabis might last for 24 h following use (78). Significant impairment of performance on a flight simulator was reported in two studies (79, 80). Research with driving simulators, however, has not found evidence for impairment extending beyond the first few hours following consumption [e.g., (39, 41)], consistent with measures of subjective effects of the drug. Other investigators have concluded that impairment is linked to blood THC levels, and is highest in the initial period after cannabis use and declines in the few hours after consumption when blood THC levels typically drop below 3–5 ng/ml (81, 82). The development of Lower Risk Guidelines for Cannabis Use at the Centre for Addiction and Mental Health in Toronto, Canada, based on these observations, recommended that an individual who has smoked one cannabis cigarette wait at least 6 h before driving, or longer if feelings of intoxication remain (83).

## Concluding Comments

As more jurisdictions move toward legalization of cannabis, regulators are likely to be increasingly concerned with the road safety impact of these changes and how any negative effects can be attenuated or avoided. While in the past there has been controversy over whether cannabis use and DUIC presented road safety risks, more recent research provides converging evidence that DUIC can increase collision risk and may be an important contributor to deaths and injuries resulting from collisions. Young adults appear most likely to engage in DUIC. Acute effects of cannabis on driving-related behaviors may include an increase in weaving, and a reduction in speed. Effects on reaction time have also been reported. This seems true regardless of the route of administration although more research is needed. At present, all studies of the effects of oral cannabis on driving consisted of synthetic THC (dronabinol) and no studies of cannabis edibles have been published. Evidence also exists to identify levels of cannabis in the blood at which impairment is observed and which thus may be proposed as *per se* levels for legal initiatives to deter DUIC. Nevertheless, important questions remain to be answered. Currently, little is known about the types of collisions most likely to involve cannabis, or if cannabis affects injury severity. More research is needed to understand sex differences in the effects of cannabis. Other questions include the comparative pharmacology of different modes of administration of THC (for example, are the effects of smoked cannabis and edible cannabis the same?) and different doses of THC and the extent to which regular or frequent uses may develop and display tolerance to the impairing effects of cannabis on driving behavior. As well, more investigation of the potential impairing effects of cannabis on therapeutic users is also warranted. Important questions still remain as to the duration of the effects of cannabis on driving and the time course of safe use of cannabis. Time course studies are especially important for different routes of administration that may have different pharmacokinetics.

## Author Contributions

BB, PDC, and RM: conceptualization, methodology, and writing—review and editing. PDC: writing—original draft preparation. BB: project administration. All authors have read and agreed to the published version of the manuscript.

## Conflict of Interest

The authors declare that the research was conducted in the absence of any commercial or financial relationships that could be construed as a potential conflict of interest.

## Publisher's Note

All claims expressed in this article are solely those of the authors and do not necessarily represent those of their affiliated organizations, or those of the publisher, the editors and the reviewers. Any product that may be evaluated in this article, or claim that may be made by its manufacturer, is not guaranteed or endorsed by the publisher.
